# Respiratory health effects of exposure to low levels of airborne endotoxin – a systematic review

**DOI:** 10.1186/s12940-018-0360-7

**Published:** 2018-02-08

**Authors:** Azadèh Farokhi, Dick Heederik, Lidwien A. M. Smit

**Affiliations:** Institute for Risk Assessment Sciences (IRAS), Utrecht University, P.O. Box 80.178, 3508TD Utrecht, The Netherlands

**Keywords:** Endotoxin, Exposure, Lung function, Respiratory health, Environment

## Abstract

**Background:**

Elevated endotoxin levels have been measured in ambient air around livestock farms, which is a cause of concern for neighbouring residents. There is clear evidence that occupational exposure to high concentrations of airborne endotoxin causes respiratory inflammation, respiratory symptoms and lung function decline. However, health effects of exposure to low levels of endotoxin are less well described. The aim of this systematic review is to summarize published associations between exposure to relatively low levels of airborne endotoxin and respiratory health endpoints.

**Methods:**

Studies investigating respiratory effects of measured or modelled exposure to low levels of airborne endotoxin (average < 100 EU/m^3^) were eligible for inclusion. In total, 1362 articles were identified through a Pubmed database search, of which 31 articles were included in this review. Studies were included up to February 2017. Overview tables and forest plots were created, and study quality was assessed.

**Results:**

Twenty-two included studies had a cross-sectional design, others were designed as longitudinal observational (*n* = 7) or experimental (*n* = 2) studies. Most studies (*n* = 23) were conducted in an occupational setting, some involved domestic or experimental exposure. Several studies reported statistically significant effects of exposure to low levels of endotoxin on respiratory symptoms and lung function. However, considerable heterogeneity existed in the outcomes of the included studies and no overall estimate could be provided by meta-analysis to quantify the possible relationship. Instead, a best evidence synthesis was performed among studies examining the exposure-response relationship between endotoxin and respiratory outcomes. Significant exposure-response relationships between endotoxin and symptoms and FEV_1_ were shown in several studies, with no conflicting findings in the studies included in the best evidence synthesis. Significantly different effects of endotoxin exposure were also seen in vulnerable subgroups (atopics and patients with broncho-obstructive disease) and smokers.

**Conclusions:**

Respiratory health effects of exposure to low levels of airborne endotoxin (< 100 EU/m^3^) seem plausible. Future studies are needed to investigate ambient exposure to endotoxin and potential respiratory health effects, especially in vulnerable subgroups of the population.

**Electronic supplementary material:**

The online version of this article (10.1186/s12940-018-0360-7) contains supplementary material, which is available to authorized users.

## Background

Health effects of air pollution have mainly been studied in urban areas, where pollutant concentrations can be high due to emissions from industries and traffic. However, poor air quality in rural areas may also be of influence on people’s health. In the Netherlands, regions where air quality is influenced by emissions from livestock farms are densely populated [1, 2]. Since potential health effects of these emissions are relevant to all people living and working in these areas, the relationship between exposure and health is a current topic of research.

Over the last thirty years, a considerable amount of research has been performed to gain insight into the respiratory health risks of people occupationally exposed to high concentrations of organic dust and endotoxin [3–6]. Inhalation of endotoxin, a lipopolysaccharide component of the cell-wall of Gram-negative bacteria present in organic dust, induces an inflammatory response in the lungs [6–9]. Aerosolized endotoxin is absorbed onto the surface of particulate matter and thus transported through the air [7, 10]. By binding to the CD14/TLR4/MD2 receptor complex on macrophages it triggers the production of cytokines and proteins that cause inflammation [8, 9, 11]. When challenged with aerosolized endotoxin, people have shown a hundredfold increase in neutrophil levels and tripling of lymphocyte levels in bronchoalveolar fluid [12]. In 1987, Castellan et al. found a clear exposure–response relationship between endotoxin concentration and group mean percentage change in forced expiratory volume in one second (FEV_1_) in individuals experimentally exposed to endotoxin containing cotton dust [13]. The effects of exposure to endotoxin are predominantly respiratory, including decline in lung function and increased prevalence of chronic bronchitis and asthma-like syndrome [5, 14, 15]. In addition to adverse health effects, occupational endotoxin exposure in agricultural workers has also been implicated in protective effects on allergic sensitisation and hay fever [16, 17].

While respiratory health effects of exposure to high levels of endotoxin are well described, potential effects associated with low levels of exposure are less well established. However, interest in the possible adverse health effects of endotoxin exposure on non-occupationally exposed populations is growing [2, 18]. Ambient endotoxin concentrations in the proximity of livestock farms and bioaerosol levels near composting sites have been found to be in the lower range of exposure levels measured in several occupations [19, 20]. Since it is not clear whether effects observed at high exposure levels can be extrapolated to lower exposure levels, further research is warranted. These outcomes are interesting for governmental institutions in particular, in order to formulate guidelines to protect the public health and safety of their inhabitants. Currently, the Dutch Expert Committee on Occupational Safety (DECOS) of the Health Council recommends a health-based occupational exposure limit of 90 EU/m^3^ [21]. DECOS regards an exposure level of 90 EU/m^3^ as a NOEL (no observed effect level), based on the effects on FEV_1_ of six-hour exposure to endotoxins in the study by Castellan et al. [13]. Based on the occupational exposure limit, a tentative limit of 30 EU/m^3^ was recommended for the general population living in the surroundings of livestock farms [21, 22].

The aim of this systematic review is to investigate the possible respiratory health effects of exposure to low levels of airborne endotoxin in humans. Levels up to 100 EU/m^3^ are included since these levels can be compared to peak ambient levels of airborne endotoxin in livestock-dense areas [23, 24] We hypothesize that exposure to these concentrations of endotoxin can have modest, but negative effects on respiratory health.

## Methods and design

### Design

This systematic review was performed by the first author (A.F.) in collaboration with the last author (L.A.M.S) and was performed according to the steps of the PRISMA statement [25].

### Information sources and search strategy

The Pubmed database was searched for relevant literature published until February 14th 2017. Search terms used to find eligible articles were based on the terms endotoxin, exposure, lung function and respiratory symptoms (such as cough, wheeze, chest tightness and shortness of breath). The full electronic search query is presented in Additional file 1: Supplement 1. Reference lists of all included studies and relevant literature reviews were searched for additional eligible articles.

### Inclusion criteria

Studies were eligible for inclusion if measurements of airborne endotoxin concentrations were performed, through either active or passive air sampling methods. Studies which used modelling approaches based on air exposure measurements were also included. Respiratory outcomes (lung function measurement and/or respiratory symptoms) had to be defined and described. Only human experimental or observational studies were included, with full text written in English, Dutch, German or French and which were originally published in peer-reviewed journals. Case reports, literature reviews and non-human studies were excluded. Also studies with measurements of airborne endotoxin of only high levels of exposure (an average of >100 EU/m^3^) were excluded, as were studies where endotoxin was measured in dust reservoirs only. The exposure variable of interest was exposure to low levels of endotoxin (average < 100 EU/m^3^). The main outcome was the effect on respiratory health; both on pulmonary function and occurrence of respiratory symptoms (coughing, wheezing, shortness of breath, asthma, dyspnoea).

### Study selection

Assessment of manuscripts for meeting the inclusion criteria was performed in a Mendeley database. Duplicates were removed and subsequently studies were selected based on title or abstract for full text-screening. In case a study was excluded based on full text screening, the reason for exclusion was listed. In case several publications reported measurements from the same series, the one with the most detailed methodology description and original values was included.

### Data extraction

Extraction of data was performed systematically by summarizing information on author, publication year, country, study design, endotoxin measurement techniques, spirometry measurements, questionnaires and confounders in overview tables.

Studies were categorized according to characteristics of the sample population (i.e., occupationally exposed subjects, respiratory disease patient groups or general population). Results are presented by individual study, since the studies were too heterogeneous in terms of endpoint measurement and presentation of endotoxin exposure levels, population samples, settings, reported outcomes and data analysis techniques to compare the results. Therefore, a narrative synthesis was performed and a best evidence synthesis was conducted for suitable outcome variables (see data synthesis). Also, forest plots were constructed (using R, version 3.3.2) to improve readability and comparability of the results.

### Methodological quality and risk of bias

Assessing quality of evidence and risk of bias in individual studies was performed using the NIH Quality Assessment Tool for Observational Cohort and Cross-Sectional Studies [26]. This tool was designed to assess the methodological quality of cohort and cross-sectional studies. In this method, the quality of the studies is evaluated by rating fourteen items representing research question, study population and sample size, participation rate, timeframe, variation in exposure level, validity and reliability of exposure and outcome variables, blinding, loss to follow-up and confounding. Each item can be scored as ‘yes’, ‘no’, ‘not applicable’, ‘cannot determine’ or ‘not reported’. The overall scores of the different studies were presented as percentages to improve comparability. Studies with total scores ≥90% were considered strong, studies scoring 70–90% were considered of moderate quality and studies scoring below 70% were considered weak.

### Data synthesis

Because of the heterogeneity of the included studies we refrained from performing a meta-analysis, but conducted a best evidence synthesis to come to some overall conclusions using the method described by Proper [27]. Only those articles that investigated the exposure-response relationship between endotoxin exposure and an outcome variable were included in the best evidence synthesis. Four outcome variables were selected for inclusion in the best evidence synthesis: wheeze, cough, (nocturnal) asthma symptoms and FEV_1_. Other reviews that applied this best evidence synthesis method considered results to be consistent when at least 75% of the studies showed statistically significant results in the same direction (defined according to *p* < 0.05) [27–29]. Originally three possible levels of evidence followed from this best evidence synthesis method, namely strong, moderate and insufficient evidence. In our evidence synthesis, we added the category ‘weak evidence’ in case the results could not be considered consistent according to Proper (not meeting the criterion of at least 75% significant results), but all studies showed results in one direction, of which at least two studies with significant results, and no conflicting findings existed for an outcome variable.

## Results

### Study selection

**Fig. 1:**
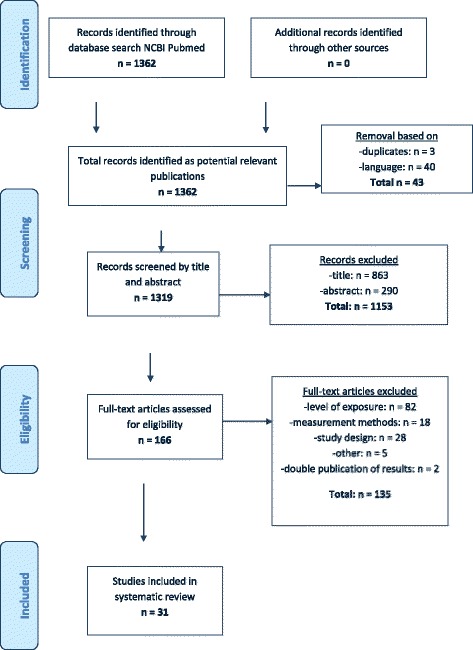
PRISMA Flow Diagram

The search yielded a total of 1362 articles. In Fig. 1, a PRISMA flowchart of the study selection is presented. After removal of duplicates (*n* = 3) and selection on language (*n* = 40), 1319 articles remained. In total, 1153 articles were excluded based on title and abstract, leaving 166 articles to be assessed by screening the full text. Most of these articles were excluded because levels of exposure (*n* = 82), endotoxin level measurement techniques (*n* = 18) or study design (*n* = 28) did not match the inclusion criteria. Two studies were removed due to duplicate publication of the same endotoxin and outcome data [30, 31]. Reference lists of all included studies and 11 relevant literature reviews were searched for additional eligible articles, but did not yield additional studies for inclusion. In total, 31 articles were included in this systematic review.

### Characteristics of included studies

#### Setting and population

An overview of the characteristics of the included studies is presented by publication date in Tables 1 and 2. Of the studies that were included most had a cross-sectional design, seven studies were set up longitudinally (with follow up periods between 5 days [32] and 11 years [33]) and two experimental studies [34, 35] were included. Eleven studies were performed in the United States, four in the Netherlands, three in Norway, two in Denmark, two in Switzerland, two in Sweden and one each in Australia, New Zealand, Germany, Canada, Pakistan, Poland and the UK. The included studies were performed between 1987 and 2016. Most of the studies examined endotoxin exposure among occupationally exposed subjects (*n* = 23), such as workers in wood, sewage and textile industries. Four studies focussed on susceptible populations, mostly children with asthma or adults with COPD. The remaining studies included children as a target population, except for one of the experimental studies where healthy adults were studied [35]. The number of included subjects ranged from 22 [36] to 3867 [37] subjects. In some cases the study was initiated because of specific reasons, such as a sudden increase in incidence of specific complaints reported by a group of workers. In some of these studies, other air pollutants than endotoxin were measured as well. We summarized important relationships between the other airborne agents and respiratory outcomes in Additional file 1: Supplement 2.

**Table 1:** Study characteristics of studies with non-occupational populations

*Non-occupational populations*								
Author (Year) Country	Study design^a^	Population	Age^b^	Endotoxin assessment methods	Exposure variables measured	Spirometry	Questionnaire	Confounders accounted for
*Asthma/COPD patient populations*								
Rabinovitch et al. (2005) US [45]	CS	24 asthmatic school-children	8.8–9.0 (SD 1.0–1.1)	-Personal and stationary sampling -37 mm Teflon filter - 2 L/min -LAL assay	Airborne endotoxin, PM	FEV_1_	Questionnaire on asthma severity	–
Delfino et al. (2015) US [46]	L: follow up 10 days	43 asthmatic school-children	14.3 (range 9–18)	-Personal sampling (10 days) - 2.5 μm cyclone filter -4 L/min -LAL assay	PM_2.5_, endotoxin	FEV_1_ and FeNO	Reporting the use of asthma medication and symptoms every two waking hours	Temperature, humidity
Lai et al. (2015) US [47]	L: follow up 12 months	248 asthmatic school-children	8 (range 4–13)	-Area air sampling with charged particle samplers (Quadra) -LAL assay	Airborne endotoxin, settled dust and settled endotoxin	FEV_1_ and FVC	Questionnaire on respiratory symptoms and follow-up phone calls	Age, sex, race, annual income, controller medication, home and school settled dust endotoxin and mouse allergen, season
Bose et al. (2016) US [62]	L: follow up 6 months	84 COPD patients	68.9 (SD 7.4)	-Area air sampling -37-mm Teflon filters -4 L/min -LAL assay	Airborne and dust endotoxin, PM, second hand smoke, NO_2_	FEV_1_	Combination of MRC dyspnoea scale, modified ATS-DLD, and St. George’s Respiratory Questionnaire	Age, gender, education, season of sampling, baseline pre-bronchodilator percent predicted FEV_1_
*Other non-occupational populations*								
Schiffman et al. (2005) US [35]	E: 1 h expo-sure	48 healthy subjects	26 (±9.46)	- Air sampling -Fiberglass filters -46 L/min -LAL assay	H_2_S, ammonia, total suspended particles, endotoxin and odour	FVC, FEV_1_and FEF_25–75%_	Environmental Exposures and Health Questionnaire	Subjects were their own controls.
Hoopmann et al. (2006) Germany [37]	CS	3867 children	Range: 5–6	Individual exposure estimated with Lagrange dispersion model based on the emission of neighbouring livestock facilities^c^	Airborne endotoxin, fungi, bacteria, total dust	–	Questionnaire of the ISAAC studies for respiratory and allergic symptoms	Atopic status parents
Horick et al. (2006) US [38]	L: follow up 12 months	360 children	range 2–3 months	Airborne endotoxin levels were calculated from dust endotoxin levels^d^	Endotoxin, dust	–	Monthly telephone calls during the first year of the child’s life	Race, pets, total mass of dust collected, concrete floor, water damage and respiratory illness
Ramagopal et al. (2014) US [61]	CS	75 children	Range: 3–59 months	Method 1: Stationary Indoor Monitors (SIM) Method 2: PIPER (pre-toddler inhalable particulate environmental robotic)	PM, endotoxin	–	ISAAC questionnaire	Age, gender, family history of asthma, floor covering, pets

^a^*CS* cross-sectional, *L* longitudinal, *E* experimental

^b^Age (years): mean, presented as range if mean age is presented for subgroups only

^c^Emissions used in the dispersion model were measured in the surroundings of stables. Meteorological data and data on the amount and type of animals were included in the model and exposures were assigned to participants’ address coordinates

^d^A measurement error correction analysis was performed according to the regression calibration method. Initial measurements were endotoxin levels derived from living-room floor dust. Regression calibration was performed using 93 living-room airborne endotoxin measurements (polycarbonate filters, 2 L/min)

**Table 2:** Study characteristics of studies with occupational populations

*Occupational populations*									
Author (Year) Country	Study design^a^	Population	Age^b,c^	Endotoxin assessment methods	Exposure variables measured	Duration of exposure^d^	Spirometry	Questionnaire	Confounders accounted for
Kawamoto et al. (1987) US [48]	CS	128 cotton workers	35.6–38.6 (±2.47–2.90)	-Area sampling -breathing zone height -LAL assay	Total dust, endotoxin, oil mist and boric acid	6.3–13.1 (±1.28–2.03)	FVC and FEV_1_	Standardized questionnaire	Age, smoking
Kateman et al. (1990) Netherlands [32]	L: follow up 5 days	40 textile yarn workers exposed to spray-humidifier, 42 controls exposed to other or no humidifier	30	-Personal sampling -Glass fibre filter - 2.0 L/min -LAL assay	Dust, endotoxin, fungi, bacteria	–	FVC, FEV_1_, PEF, MEF_75_, MEF_50_, MEF_25_, MMEF	Modified MRC questionnaire	Smoking, age, height, standing height
Dahlqvist et al. (1992) Sweden [52]	CS	28 wood trimmers, 19 office workers (controls)	S: 37 (±11), C:42 (±8)	-Personal sampling -Millipore filter - 2 L/min -LAL assay	Dust, endotoxin, moulds and bacteria, terpenes	13 (±11)	FEV_1_ and FVC, MEF_50_, MEF_25_	Modified MRC questionnaire	Smoking
Sprince et al. (1997) US [54]	CS	183 machine workers in automobile industry, 66 assemblers (controls)	43.2–43.7 (± 7.6–8.4)	-Area and personal sampling -MCE filters -LAL assay	MWF, total aerosol, endotoxin, total fungi, total bacteria, total organisms	S: 12 (±9) years, C: 8 (±8) years (*p* = 0.023)	FEV_1_ and FVC	Modified ATS-DLD questionnaire	Smoking status, gender, age, race
Zock et al. (1998) Netherlands [39]	CS	57 potato processing workers	39–41 (±7–10)	-Personal sampling -Glass fibre filter - 2.0 L/min -LAL assay	Endotoxin exposure	13–14 years (± 7–9)	FVC, FEV_1_, MMEF and PEF	MRC questionnaire	Smoking
Mandryk et al. (1999) Australia [53]	CS	168 wood workers, 30 maintenance workers (controls)	S: 37 (± 12.8), C:39 (±11.7)	-Personal sampling -Polycarbonate filter 25 mm -LAL assay	Airborne dust, endotoxin, micro-organisms, (1- > 3)-B-D-glucan	11 (±10.6 years)	VC, FEV_1_ and FVC	Combination of the Organic Dust Questionnaire and the MRC respiratory questionnaire	Age, height, smoking, number of years of exposure
Mahar et al. (2002) UK [40]	L: follow up 5 years	87 refuse derived fuel plant workers	39.9–41.4 (SD 8.0–8.1)	-Personal and area sampling -0.45 μm endotoxin free filters -LAL assay	Airborne endotoxin	7.7–11 years (SD 5.4–5.9)	FEV_1_ and FVC	Questionnaire	Smoking, length of employment
Wouters et al. (2002) Netherlands [59]	CS	47 waste collecting workers, 15 controls	S:34.0 (SD 10.2) C: 36.4 (SD 6.4)	-Personal sampling -Glass fibre filter -3.5 L/min -LAL assay	Dust, endotoxin, β(1-- > 3)-glucan	Median 5 years	–	Modified MRC questionnaire	-
Fransman et al. (2003) New Zealand [55]	CS	112 plywood mill workers, 415 controls of the general population	S: 34.5 (±9.1), C: 32.5 (±7.2)	-Personal sampling -Glass fibre filter -2.0 L/min -LAL assay	Inhalable dust, endotoxin, abietic acid, terpenes, formaldehyde	4.7 years (±3.5)	–	Combination of ISAAC, MRC and ECRHS questionnaires	Age, gender, ethnicity
Heldal et al. (2004) Norway [36]	CS	22 waste collection workers	AM: 32 (range 20–62)	-Personal sampling -25 mm polycarbonate filter -2 L/min -LAL assay	Total dust, endotoxin, bacteria, fungal spores	–	–	Questionnaire	Age, smoking
Kennedy et al. (2004) Canada [34]	E^e^: follow up 1 month	226 glass bottle recycling workers, 212 ferry workers (controls)	S: 41.2–45.5 (±8.4–9.0)	-Personal sampling -2 L/min -LAL assay	Endotoxin, dust, fungi	12.0–17.3 years (±7.4–8.4)	–	Modified ATS questionnaire	Age, gender, race, smoking status, history of hay fever and asthma
Sigsgaard et al. (2004) Denmark [33]	L: follow up 11 years	97 paper mill workers, 55 water-supply workers (controls)	39–43 (±7–9)	-Personal sampling - 37 micropore filters -LAL assay	Total dust, endotoxin, micro-organisms	–	FVC and FEV_1_	–	Age, smoking, atopy
Smit et al. (2005) Netherlands [41]	CS	371 waste water workers; 97 office staff, 2698 general population members (controls)	43.4–47.3 (± 9.9–10.1)	-Personal sampling -Glass fibre filter - 3.5 L/min -LAL assay	Endotoxin	12.6–14.9 years (±9.0–10.2)	–	Questionnaire specifically developed for bioaerosol related health effects in waste recycling and composting industry	Age, gender and smoking habits
Widmeier et al. (2007) Switzerland [42]	CS	409 wastewater-and garbage workers, 369 public transport and forestry workers (controls)	median 41–47 (5th–95th% 22–58)	-Personal and area sampling -LAL assay	Endotoxin exposure	–	FVC and FEV_1_	Questions from SAPALDIA-study questionnaire	Age, gender, height, packyears, BMI, serum creatinine, job change, smoking history
Rusca et al. (2008) Switzerland [60]	CS	111 sawmill workers	group 1: 26.5 (±9.5), group 2: 36.7 (±8.5), group 3: 47.7 (±8.2)	-Area sampling -Glass fibre filter - 2.0 L/min -LAL assay	Airborne dust, endotoxin, fungi and bacteria	group 1: 1–5 years, group 2: 5–20 years, group 3: > 20 years	FVC and FEV_1_	Combination of the Organic Dust Questionnaire and the MRC respiratory questionnaire	Height and packyears of cigarette smoking
Dang et al. (2010) US [49]	CS	69 water resort workers, 74 office workers (controls)	S: 20 (range 16–50), C: 31 (range 15-61)	-Area sampling -Polyvinyl-chloride filters - 2 L/min - LAL assay	Chloramines, endotoxin, Legionella, Mycobacterium	2.8 months	–	Questionnaire	Smoking, asthma status
Renström et al. (2011) Sweden [50]	CS	59 pet shop workers	31.4 (±9.9)	-Personal sampling -1 μm filter - 2.0 L/min -LAL assay	Endotoxin and aeroallergens	9.4 (±7.4)	VC, FVC and FEV_1_	Questionnaire	Smoking, gender,
Schlünssen et al. (2011) Denmark [57]	CS	232 woodchip and straw workers, 107 workers in oil/gas power plants (controls)	S: 45.9–47.7(SD 8.6–9.3), C:48.1 (SD 10.0)	-Stationary air sampling -Teflon filters - 1.9 L/min -LAL assay	Endotoxin, total dust and fungi	7–10.9 years	FVC and FEV_1_	Modified ECRHS questionnaire	Gender, height, weight, atopy, age and smoking
Meza et al. (2013) US [56]	CS	183 aircraft workers exposed to MWF, 224 office workers (controls)	S: 95% > 45 C: 92% > 45	-Area air sampling - 0.45 μm filter -2 L/min -LAL assay	Metal working fluids, endotoxin, bacteria	–	–	Questionnaire based on the ECRHS	Age, gender, smoking status and hours worked per week
Shiryaeva et al. (2014) Norway [58]	CS	70 salmon processing workers	40.1 (±11.5)	-Personal sampling -Glass fibre filter - 2.5 L/min -LAL assay	Endotoxin, parvalbumin and total protein	–	FEV_1_	Modified MRC questionnaire	Age, gender, asthma, smoking and height
Cyprowski et al. (2015) Poland [43]	CS	78 sewage workers	43	-Personal sampling -25 mm glass-fibre filter -2 L/min -LAL assay	Endotoxin in inhalable dust	8.5 years	FEV_1_ and FVC	–	Inhalable dust, smoking habits
Heldal et al. (2015) Norway [51]	CS	47 compost workers, 37 office controls	S: 41–42 (± 9–11), C: 43 (±10)	-Personal sampling -Glass fibre filter - 2.0 L/min -LAL assay	Dust, endotoxin, bacteria, fungal spores, actinomycetes	–	FEV_1_/FVC and FEV_1_	Questionnaire	Smoking packyears, age, atopy
Ghani et al. (2016) Pakistan [44]	CS	100 textile mill workers, 100 controls	3 groups: < 30, 30–40, > 40	-Area air sampling -Glass fibre filter -LAL assay	Airborne endotoxin	≥5 years	FEV_1_, FVC, FEV_1_/FVC, PEF	Modified ATS questionnaire	Age, duration of exposure

^a^*CS* cross-sectional, *L* longitudinal, *E* experimental

^b^*S* exposed subjects, *C* controls

^c^Age: mean, presented as range if mean age is presented for subgroups only

^d^Presented as mean, unless stated otherwise

^e^Half of the participating shops shut down glass-breaking activity during this experiment

#### Measured pollutants

Dust was mostly collected with personal sampling techniques during working hours, alternatives used were area sampling and predictive calculations based on dispersion models [37, 38]. Endotoxin was measured using the Limulus Amebocyte Lysate (LAL) assay, which is the most accepted assay for endotoxin exposure measurements. The exposure agents measured in the included studies vary greatly. Some studies only reported measurement of the exposure to airborne endotoxin [39–44] whereas other studies included measurement of dust, bacteria, fungi and/or other airborne particles.

#### Health outcomes

Twenty-one studies performed spirometry measurements and included lung function values in their design, most of them included FEV_1_ and FVC as outcome measurements. All but three studies recorded symptoms through a questionnaire. Questionnaires used were often based on questions from the MRC, ATS, ECRHS or ISAAC questionnaires and the Organic Dust Questionnaire, but a number of other questionnaires were used as well as a source for the reporting of symptoms [35, 36, 40–42, 45–51]. The study by Horick et al. used monthly telephone calls to register respiratory symptoms [38].

### Quality assessment

An overview of the quality assessment results is presented in Additional file 1: Supplement S3. All studies had well-described study objectives and most included detailed information on the study subjects. The main reason for scoring negative on study population description was the absence of the inclusion period. Eleven studies did not report the participation rate. All but one studies reported effect sizes, only Heldal et al. presented results otherwise [36]. Since most of the studies had a cross-sectional design, exposures were not measured prior to the outcomes. In case studies included cross-work shift or cross-week measurements of lung function values and in case of longitudinal studies, the item timeframe was scored positive. Twenty studies investigated effects of different levels of exposure. All studies used valid and reliable area or personal exposure measurements. Exposure assessment over time was scored positive when repeated personal full-work shift measurements were performed, area measurements were considered insufficient. Two studies used modelling to estimate personal endotoxin exposure, this was also regarded an accurate and reliable way of estimating exposure [37, 38]. Regarding outcome measures, spirometry measurements were considered valid and reliable, as was the use of validated questionnaires. Dang et al. were the only ones using an unvalidated questionnaire without performing additional spirometry measurements [49]. Blinding of outcome assessors was only applicable in non-occupational studies, as is reflected in the scoring of this criterion. Four studies did not perform/report correction for confounding. Since not all scoring items were applicable for all included studies, a percentage of the maximum score was calculated for each included study. The percentages of total scores varied between 55% and 100%. Most studies were considered moderate based on their score (*n* = 13), others were considered strong (*n* = 9) or weak (*n* = 9).

### Findings

Main results of questionnaires, spirometry and dose-response relationships are combined and summarised in Table 3.

**Table 3:** Summary of main results on airborne endotoxin exposure and respiratory outcomes assessed by questionnaires and spirometry

Results			
Author (year)	Population	Levels of airborne endotoxin exposure (EU/m^3^)	Conclusion
*Non-occupational populations*			
Rabinovitch et al. (2005) [45]	24 asthmatic school-children	Interval 1: median 0.08 EU/m^3^, IQR 0.09	Higher personal endotoxin exposure was significantly related to more sleep-related asthma complaints and decreased evening FEV_1_ in children with asthma in a dose-dependent manner.
		Interval 2: median 0.37 EU/m^3^, IQR 0.16	
Schiffman et al. (2005) [35]	48 healthy subjects	7.40 EU/m^3^	No statistical significant effects of the 1 h exposure to endotoxin on lung function changes or respiratory symptoms.
Hoopmann et al. (2006) [37]	3867 children	Median 0.064 EU/m^3^, IQR 0.025–0.141^a^	Increase of asthmatic symptoms and wheezing due to exposure to airborne endotoxin was significant for children of atopic parents.
Horick et al. (2006) [38]	360 children	Mean 0.81 EU/m^3^, range 0.23–5.87	Exposure to airborne endotoxin leads to a significant increase in prevalence of wheeze.
Ramagopal et al. (2014) [61]	75 children	SIM: median 0.6 EU/m^3^, range 0.03–8.6	No significant differences in prevalence of wheeze or asthma symptoms among children exposed to different levels of endotoxin.
		PIPER: median 1.0 EU/m^3^, range 0.09–16	
Delfino et al. (2015) [46]	43 asthmatic school-children	Mean 2.04 EU/m^3^ (±3.71), range 0.002–25.3	Personal endotoxin exposure was not associated with acute daily changes in FEV_1_ overall. Among patients with %predicted FEV_1_ < 80%, however, daily FEV_1_ significantly decreased with increase in personal endotoxin exposure with 2.19 EU/m^3^.
Lai et al. (2015) [47]	248 asthmatic school-children	GM 24.7 EU/m^3^, range 0.2–780.0 (of which 78% < 90 EU/m^3^)	In subjects who were non-atopic, higher concentrations of school air endotoxin were significantly associated with increased daytime wheeze, exercise related symptoms and maximum symptom days in a dose-dependent manner.
Bose et al. (2016) [62]	84 COPD patients	Mean 0.55 EU/m^3^ (±1.3)	No significant associations between airborne endotoxin and increased respiratory/COPD morbidity.
*Occupational populations*			
Kawamoto et al. (1987) [48]	128 cotton workers	3 groups: < 17 EU/m^3^, 17–117 EU/m^3^ and > 117 EU/m^3a^	A non-significant decrease in FEV_1_ was seen in workers exposed to more than 17 EU/m^3^.
Kateman et al. (1990) [32]	40 textile yarn workers exposed to spray-humidifier, 42 controls exposed to other or no humidifier.	GM 0.64 EU/m^3^ (GSD 0.016) for cold-water humidification area, 0.18–0.19 EU/m^3^ for other areas.	Significant cross-work shift decreases in multiple lung function variables were found for the workers in the cold-water humidification area. Also, a decrease in multiple lung function variables was visible over the week for these workers.
Dahlqvist et al. (1992) [52]	28 wood trimmers, 19 controls (office workers)	15–25 EU/m^3^^a^	Significantly higher prevalence of dry cough, cough with phlegm and breathlessness among exposed workers. Wood trimmers seropositive for precipitating antibodies to moulds showed a significant decrease in FEV_1_ over a workweek. Subjects with a period of employment > 18 years had a significantly larger change in MEF over the workweek than subjects employed < 6 years.
Sprince et al. (1997) [54]	183 machine workers in automobile industry. 66 assemblers (controls).	GM 31 EU/m^3^, range 2.7–984	Significant difference in prevalence of cough and work-related chest tightness between exposed subjects and controls. Usual phlegm showed a significant association with increasing endotoxin exposure. No significant associations between endotoxin and change in lung function.
Zock et al. (1998) [39]	57 potato processing workers	AM 32.9 EU/m^3^. Low exposed group: AM 21 EU/m^3^, high exposed group: 56 EU/m^3^	Significant larger across-work shift decreases in lung function variables were found in subjects exposed to higher levels of endotoxin exposure when compared to lower exposed subjects.
Mandryk et al. (1999) [53]	168 wood workers. 30 maintenance workers (controls).	Inhalable endotoxin: GM 24.1–43.0 EU/m^3^(GSD 15.5–47.7)^a^	Significant differences in prevalence of respiratory symptoms, lung function decline and cross-work shift lung function changes in exposed wood workers when compared to controls. Also significant dose-response relationships between lung function decline/cross work shift changes and personal exposure to airborne endotoxin.
Mahar et al. (2002) [40]	87 refuse derived fuel plant workers	1995: GM 28.5 EU/m^3^ (GSD 2.77)	Pulmonary function values of the exposed are within predicted values and show no decrements over time for the workforce as a whole. No trends indicating reductions in lung functions based on length of employment.
		2000: GM 28.1 EU/m^3^ (GSD 6.65)	
		Total: GM 28.4 EU/m^3^ (GSD 3.75)	
Wouters et al. (2002) [59]	47 waste collecting workers. 15 office workers (controls).	GM 39.4 EU/m^3^, range 4–7182	No significant differences in prevalence of respiratory symptoms among exposed workers when compared to controls.
Fransman et al. (2003) [55]	112 plywood mill workers. 415 controls of the general population.	GM 23.0 EU/m^3^ (GSD 2.8)	Shortness of breath and wheezing were significantly more prevalent among subjects exposed to dust, endotoxin, terpenes and formaldehyde and also significantly more present in workers employed > 6.5 years (all *p* < 0.05). No clear associations between prevalence of symptoms and exposure to endotoxin alone. 53–83% of respiratory symptoms lessened during holidays.
Heldal et al. (2004) [36]	22 waste collection workers	AM 2.5 EU/m^3^, range 0–7.8	No significant difference in exposure level to endotoxin between subjects with and without respiratory complaints.
Kennedy et al. (2004) [34]	226 glass bottle recycling workers. 212 ferry workers (controls).	GM 3.6–4.3 EU/m^3^, range < 0.14–179	Significantly higher prevalence of chest tightness in the exposed groups vs unexposed subjects. No significant increase found in respiratory symptoms related to personal endotoxin exposure > 4 EU/m^3^when compared to exposure to lower levels.
Sigsgaard et al. (2004) [34]	97 paper mill workers. 55 water-supply workers (controls).	6–69 EU/m^3^, range 6–370	No significant decrements in lung function were seen among paper recycling workers exposed to endotoxin levels below 200 EU/m^3^.
Smit et al. (2005) [41]	371 waste water workers. 97 office staff, 2698 general population members (controls).	GM 27 EU/m^3^ (GSD 3.7)	Prevalence of daily cough, shortness of breath and asthma attacks were significantly higher among exposed subjects than in the general population. No significant differences in respiratory symptoms in subjects exposed to higher levels of endotoxin when compared to lower levels. Length of employment > 20 years was significantly associated with LRT and skin symptoms.
Widmeier et al. (2007) [47]	409 wastewater-and garbage workers. 369 public transport and forestry workers (controls).	Wastewater workers: winter 8.8–29.7 EU/m^3^, summer 29.8–52.6 EU/m^3^; garbage collectors winter 3.43–8.14 EU/m^3^, summer 3.63–11.03 EU/m^3^	No significant association between endotoxin exposure and lung function or respiratory symptoms.
Rusca et al. (2008) [60]	111 sawmill workers	Range 1–24 EU/m^3^	No significant relationships between respiratory symptoms or lung function tests and exposure to dust and endotoxin.
Dang et al. (2010) [49]	69 water resort workers, 74 office workers (controls).	Mean 45 EU/m^3^, range 18–84 EU/m^3^	Workers exposed to (higher levels of) endotoxin and chloramine were significantly more likely to report work-related respiratory symptoms such as cough, wheezing shortness of breath and chest tightness than unexposed colleagues.
Renström et al. (2011) [50]	59 pet shop workers	Range 1–100 EU/m^3^	No significant difference in exposure levels of endotoxin between subjects with work symptoms compared to subjects without symptoms.
Schlunssen et al. (2011) [57]	232 woodchip and straw workers. 107 workers in oil/gas power plants (controls).	Woodchip plants: median 1.7 EU/m^3^, range 0.01–6.5	Significant association between increased asthma symptoms and endotoxin exposure to 12–294 EU/m^3^. No significant relationship between endotoxin exposure and change in lung function parameters.
		Straw plants: median 74 EU/m^3^, range 1.5–294	
		Control: median 0.9 EU/m^3^	
Meza et al. (2013) [56]	183 aircraft workers exposed to MWF. 224 office workers (controls).	Mean 1.2 EU/m^3^, range 0.42–2.7	Significantly more respiratory symptoms and asthma among workers exposed to metalworking fluids and endotoxin when compared to controls.
Shiryaeva et al. (2014) [58]	70 salmon processing workers	Monday-Thursday GM 1.39–1.65 EU/m^3^, range 0.30–29.0	Wheeze and chest tightness decreased significantly over the workweek (p < 0.05). Significant decline in cross-work shift %FEV_1_ was seen on Monday. Models relating separate respiratory variables/lung function to endotoxin exposure showed no significant associations.
Cyprowski et al. (2015) [43]	78 sewage workers	AM 38.8 EU/m^3^, range 0.63–214	Small but significant across-work shift declines in FEV_1_(*p* = 0.044) associated with endotoxin exposure, independent of organic dust concentrations or smoking habits.
Heldal et al. (2015) [51]	47 compost workers, 37 office controls.	AM 4.0–38 EU/m^3^, range 0–730	Significant association between cough and exposure to 0.7–2.7 EU/m^3^ endotoxin. Cough and one or more work-related symptoms were significantly more prevalent in the compost workers when compared to controls. The predicted FVC% measured before work was significantly lower in the compost workers as compared to controls (*p* < 0.05).
Ghani et al. (2016) [44]	100 textile mill workers, 100 controls.	Range 40–300 EU/m^3^	Significantly decreased mean lung function values amongst workers exposed to airborne endotoxin when compared to control subjects.

^a^original values were presented in article in mg/m^3^ or ng/m^3^

#### Questionnaire

Respiratory symptoms recorded by most studies included cough, wheezing, shortness of breath, chest tightness and nocturnal asthma symptoms. As presented in Additional file 1: Supplement S4.1, ten studies reported a significantly higher prevalence of respiratory symptoms among exposed subjects when compared to unexposed or lower exposed controls. The definition of exposure differed between studies, ranging from only endotoxin exposure (measured) to exposure to various bioaerosols. The symptoms that were found to be significantly more prevalent among exposed subjects were cough [41, 49, 51–54], wheeze [49, 55, 56], shortness of breath [41, 49, 52, 55], (work-related) chest tightness [34, 49, 54, 56], chronic bronchitis [53] and (work-related) asthmatic symptoms [41, 56, 57]. Fransman et al. found that plywood workers exposed to 23 EU/m^3^ endotoxin had significantly more attacks of shortness of breath with wheezing than unexposed controls and that workers employed > 6.5 years had significantly more asthma, shortness of breath and wheezing when compared to members of the general population [55]. Smit et al. also showed a significant positive association between length of employment and lower respiratory tract (LRT) symptoms [41]. Two studies found that respiratory symptoms lessened during holidays/days off; 53–83% of respiratory symptoms lessened during holidays in one study [55], another study found a PR of 2.84 (95%CI 1.56–5.18) for decline in symptoms of wheeze during holidays [56]. Shiryaeva et al. found that the highest frequency of symptoms was present on Mondays and that symptoms decreased gradually over the week, wheeze and chest tightness decreased significantly [58].

One study among textile yarn workers exposed to different kinds of humidifiers (endotoxin levels 0.18–0.64 EU/m^3^) did not find a significant difference in the reporting of symptoms among subpopulations [32]. Three other studies did not find a significant difference in prevalence of respiratory symptoms among exposed subjects when compared to controls [42, 59, 60]. Two studies did not find any difference in exposure levels of endotoxin between subjects with and without respiratory complaints [36, 50]. In the study by Zock et al. among potato processing workers, subjects exposed to 21 EU/m^3^ (AM) seemed to have more symptoms of respiratory symptoms than the group exposed to 56 EU/m^3^ (AM) [39]. Exposure to 7.40 EU/m^3^ for 1 h in an experimental setting did not significantly influence the prevalence of cough symptoms [35].

**Fig. 2:**
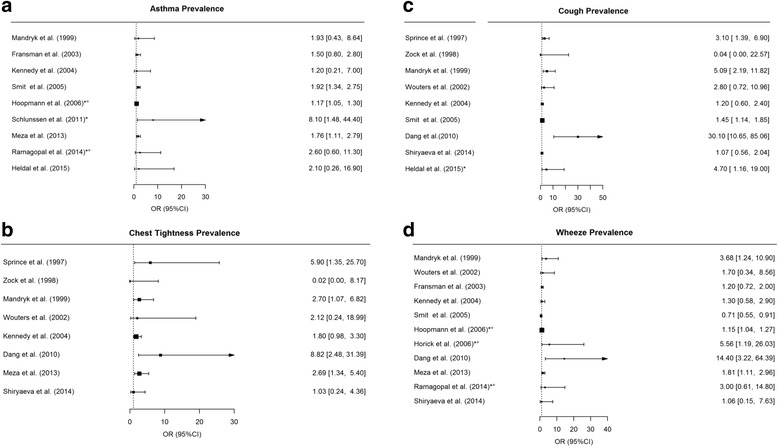
Forest plots presenting the odds ratio (OR) for asthma (a), chest tightness (b), cough (c) and wheeze (d) – exposure to multiple bioaerosols. Prevalence is calculated for exposed vs non-exposed subjects or high vs low exposed subjects. *OR for dose-dependent relationship with endotoxin (no other bioaerosols included in calculations). °non-occupational study

**Fig. 3:**
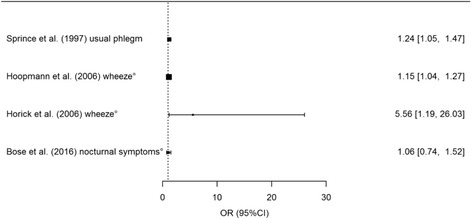
Odds Ratio (OR) for symptoms per increase in exposure of 1 log endotoxin*. *For Horick et al. (2006), the symptoms OR per increase in 0.4 log endotoxin is presented.°non-occupational study

The forest plots in Fig. 2 show a summary of the effects of exposure (to endotoxin and other bioaerosols) on respiratory symptoms presented in the included studies. The odds ratios (or exp.(beta) for symptom score) of asthma, chest tightness, cough and wheeze appear to be higher in subjects exposed to bioaerosols, albeit with wide confidence intervals and often not significant. Figure 3 shows the odds ratios for different symptoms for an increase in exposure of 1 unit of log-transformed endotoxin.

### Spirometry

The results for the different outcomes of spirometry measurements are summarised in Additional file 1: Supplement S4.2. Three studies found a significant difference in pre-shift lung function values between exposed subjects and controls (exposure definition differed between studies), where exposed subjects had lower values for FEV_1_ and/or FVC [44, 51, 53]. The baseline FVC recorded by one study was 84.7% of predicted for woodworkers exposed to 24–43 EU/m^3^ endotoxin compared to 94.9% for controls (*p* = 0.0001), for FEV_1_ comparable outcomes were found [53].

Six studies presented significant cross-work shift declines of FEV_1_ and/or FVC among exposed subjects [32, 39, 43, 46, 53, 58]. Mean absolute decrease in FEV_1_ was found to be 0.06–0.12 L among potato processing workers exposed to 56 EU/m^3^ [39], another study found an mean decrease of 0.07–0.10 L among textile yarn workers exposed to spray-humidifiers associated with endotoxin levels of 0.64 EU/m^3^ [32]. The latter study found a significant decrease in FEV_1_ over the workday but also a decreased FEV_1_ level on Friday when compared to Monday. Dahlqvist et al. found that subjects with a period of employment > 18 years had a significantly larger change in MMEF over the workweek than subjects employed < 6 years [52]. Another study that performed cross-week analyses did not find significant lung function decline over the workweek [58]. In terms of cross-work shift decline in percentage predicted lung function, one study found a cross-work shift decrease of 6.34% in FEV_1_ among woodworkers exposed to 24–43 EU/m^3^ whereas controls had a decrease of 1.78% (*p* < 0.001) [53].

Exposure to 7.40 EU/m^3^ for 1 h in an experimental setting did not lead to significant changes in lung function parameters among 48 healthy volunteers [35].

Four studies found no significant effect of exposure to bioaerosols on lung function parameters among exposed subjects [42, 48, 54, 60]. Another study among 97 paper mill workers showed no significant difference in yearly decline of lung function between low and high exposed groups (endotoxin levels ranged between 6 and 370 EU/m^3^) [33]. A longitudinal study also found no significant changes in lung function after 5 years of exposure to endotoxin levels of 28 EU/m^3^ among refuse derived fuel workers [40].

#### Dose-response relationship

Eighteen of the included studies performed analyses to study the dose-dependent exposure-response relationship between endotoxin exposure and respiratory health effects, the results are presented in Additional file 1: Supplement S4.3. Symptoms that occurred significantly more often with increasing levels of endotoxin exposure were cough [51], asthmatic symptoms [37, 45, 47, 57], wheeze [37, 38, 47] and usual phlegm [54]. One study found an OR of 2.042 (95%CI 1.029–4.042) for nocturnal asthma symptoms for every 1 EU/m^3^ increase in endotoxin exposure [45]. A relative risk of 5.56 (95%CI 1.19–26.03) for wheeze was found for every 0.4 log_10_ endotoxin increase in personal exposure in another study [38].

Five studies found a significant drop in FEV_1_ levels associated with an increase in endotoxin exposure [39, 43, 45, 46, 53]. One study found that children exposed to higher levels of endotoxin had significantly lower levels of evening FEV_1_, with a decrease of 316 ml per 1 EU/m^3^ increase (95%CI -597 to − 36 ml, *p* = 0.036) [45]. Cyprowski et al. found a 42 ml decrease in FEV_1_ per 1 EU/m^3^ increase in exposure (*p* = 0.044) [43].

#### Subgroup analyses

Wheeze, nocturnal cough and other asthmatic symptoms were more prevalent among children of atopic parents in a big German study where endotoxin exposure (median 0.064 EU/m^3^) was modelled using dispersion models. Per one log unit increase in endotoxin exposure the OR for asthmatic symptoms among children with atopic parents was 1.15 (95%CI 1.03–1.29) [37].

One study among asthmatic school children found that subjects with baseline FEV_1_ < 80% of predicted had significant associations with endotoxin exposure, predicted FEV_1_ values dropped with 7.7% (95%CI -12.3 to − 3.3%) for every 2.19 EU/m^3^ increase in exposure [46]. Another study among asthmatic school children found that airborne endotoxin was associated with increased maximum symptom-days only in subjects with non-atopic asthma. For atopics, there was an inverted U-shaped relationship between school air endotoxin and maximum symptom-days (plateau at 230 EU/m^3^) [47].

In an occupational study, 60% of exposed asthmatic workers reported that their asthma seemed worse at work, while none of the non-exposed asthmatic subjects reported this [49]. A second occupational study found that atopic exposed subjects had a significantly higher proportion with symptoms at work (PR 3.2 (95%CI 1.6–6.2), *p* < 0.001) than non-atopics [50]. Another study showed that there were no significant differences in respiratory outcomes related to exposure between atopic and non-atopic subjects [58].

In the study by Schlünssen et al., asthma symptoms were found to be associated with endotoxin in non-smokers (OR 10.1;1.7–59.7), whereas this was not found for smokers (OR 0.5; 0.1–2.8) [57]. Dahlqvist et al. found no differences in the distribution of symptoms between smokers and non-smokers [52]. In one study, non-smokers showed larger across work shift declines than smokers: for FEV_1_ the across work shift difference was − 0.1% (95%CI -3.6;3.5) for smokers and − 1.8% (95%CI -4.5,1.0) for non-smokers [39]. On the contrary, in another study smokers showed an across work shift decline of 1.12% (SD 9.5) for FVC and 2.26% (SD 12.1) for FEV_1_, whereas non-smokers showed an across work shift decline of 0.53% (SD 11.9) for FVC and 0.73% (SD 12.5) for FEV_1_ [40]. Similarly, smokers had a mean cross work shift decline in FEV_1_ of 0.93% (SD 5.24), this was 0.72% (SD 6.31) for former smokers and 0.41% (SD7.52) for non-smokers in a second study [58]. Yet another study showed comparable lung function declines for smokers and non-smokers [33].

One study found that smokers exposed to 3–11 EU/m^3^ endotoxin had significantly lower lung function values than non-exposed smokers. For ex-smokers, no significant difference was found according to exposure [42].

An overview of the results among subgroups is provided in Additional file 1: Supplement S4.4.

## Best evidence synthesis

In Additional file 1: Supplement S5, an overview of the best evidence synthesis is presented.

For wheeze, there were two strong studies and one study of moderate quality showing a significant increase in complaints when personal endotoxin exposure increased [37, 38, 47]. Four other studies also showed dose-dependent increase of wheeze, although their results did not reach significance, which may be due to limited sample sizes in some of these studies [57, 58, 61, 62]. The evidence for the effects of endotoxin exposure on wheeze symptoms could be classified as weak, since less than 75% of the results found reached significance, but none of the studies showed results in the opposite direction.

For nocturnal asthma symptoms, there were one strong study and three studies of moderate quality showing a significant dose-dependent increase of symptoms [37, 45, 47, 57]. Two other studies (in < 100 subjects) showed an increase of symptoms as well, but their results did not reach significance [61, 62]. This was considered weak evidence for effects of exposure to endotoxin on asthma complaints since less than 75% of the results reached significance. No studies mentioned evidence for improvement of asthma symptoms related to endotoxin exposure, however.

Only one study, with a design classified as weak, found a significant dose-dependent effect of exposure to endotoxin on symptoms of cough [36]. Another strong study did suggest the same effect but did not reach statistical significance [58]. On the contrary, Zock et al. found that subjects exposed to lower levels of endotoxin had a higher prevalence of cough symptoms than subjects exposed to higher levels of endotoxin [39]. Overall, insufficient evidence was found to state an effect of endotoxin exposure on symptoms of cough.

There were also several studies investigating the dose-dependent effects of exposure to endotoxin on FEV_1_ levels. Four strong studies and one study of moderate quality found significant declines of FEV_1_ (cross-work shift or cross-day) in relation to increasing endotoxin exposure [39, 43, 45, 46, 53]. Two other studies (in 70 salmon workers and 128 cotton workers) found non-significant declines in FEV_1_ with increasing endotoxin exposure [48, 58]. Overall, evidence regarding the effect of endotoxin on decline in FEV_1_ was considered to be weak since less than 75% of the results were significant, although multiple strong studies support the hypothesis that FEV_1_ declines with higher endotoxin exposure and no studies found results in the opposite direction.

## Discussion

This review systematically summarizes the current knowledge on the respiratory effects of exposure to low levels of endotoxin. To our knowledge, no previous systematic review presented health effects of exposure to airborne endotoxin at levels that can be found in polluted ambient air, for instance near large-scale livestock farms or composting sites. Overall, negative effects on lung function and an increase in respiratory symptoms seem present although the evidence found was inconsistent in several ways.

By performing a best evidence synthesis we attempted to rate the level of evidence of the results found. Through this synthesis we could conclude that there is weak evidence regarding effects of low levels of airborne endotoxin on FEV_1_ values, although multiple strong studies showed significantly decreasing FEV_1_ values related to higher endotoxin exposures. For other outcomes too, only weak or insufficient evidence was found. This was mainly due to a lack of statistically significant findings, as many studies were underpowered, in particular for studying dichotomous outcomes. Still, most of the included studies did suggest negative effects of exposure to airborne endotoxin on wheeze, cough and (sleep-related) asthma symptoms. Apart from the exposure-response associations included in the best evidence synthesis, several other studies indicated that exposure to airborne endotoxin can have respiratory effects at these levels of exposure. Overall, twelve out of eighteen studies found statistically significant dose-dependent effects of exposure to endotoxin on respiratory symptoms and/or lung function values.

### Strengths and limitations

One of the limitations concerning this review is the use of only one database in the search for relevant literature. Although Pubmed is widely used and expected to include almost all relevant literature on the topic of interest by the authors, it might be that relevant literature was not identified because of the exclusion of other databases. Another limitation is the inclusion of mostly cross-sectional observational studies and the strength of evidence must be interpreted against that background, the findings of this study remain descriptive. Further research in the field of respiratory inflammation related to endotoxin exposure at low levels would strengthen the evidence, as would investigation to certain biomarkers to prove a causal relationship. Since the nature of the included studies was quite heterogeneous and the statistical methods, sample populations and exposure and outcome definitions varied too much in the different studies, a meta-analysis, or meta-regression, could not be performed. A limitation in the assessment of the quality of the studies is the absence of clear cut-off points for considering a study design strong, moderate or weak. To overcome this, overall quality scores were compared by calculating percentages and strict cut-off points were formulated. Other limitations of the approach in this systematic review are the influences of multiple testing, selective reporting and publication bias.

A strength in the design of this review is the systematic approach and conduction of the inclusion and assessment of the relevant literature and data extraction of the included articles. By this systematic approach, chances of missing relevant literature or data was minimalized. The careful quality assessment, which was conducted by two researchers to optimise critical appraisal, is another strength of this study and improves the interpretation of the results of the different included studies. Although most of the included studies were designed cross-sectionally, several longitudinal follow-up studies were included. Inclusion of these articles gives insight in longer term changes in lung function and adds to the clinical relevance of this review. Another strength of this review is the inclusion of only actual measured levels of airborne endotoxin, enabling the nearest approximation of the true exposure of the included subjects. The only other exposure measurement method which was acceptable was modelling of personal endotoxin exposures based on measured airborne endotoxin levels. Hoopmann et al. used a dispersion model to predict personal endotoxin exposures by using endotoxin emission measurements of neighbouring livestock farms. All studies used the functional LAL assay to measure endotoxin exposure. Although within-laboratory precision of the assay is good, variation between laboratories may be substantial, in particular if different extraction and analysis procedures are used [21]. Underestimation of endotoxin levels, especially when using older protocols, may have resulted in the inclusion of studies with true mean endotoxin levels above 100 EU/m^3^, although most studies had mean exposure levels far below this threshold. The best evidence synthesis was conducted to strengthen the statements on the evidence of the reported results. Only dose-dependent exposure-response relationships were used in the best evidence synthesis in order to rely only on those results that were fully attributable to exposure to endotoxin.

### Significant respiratory effects of other airborne agents

This review aimed to summarize associations between endotoxin and respiratory health, but it should be noted that airborne endotoxin levels are generally correlated with other bioaerosol components such as fungi and bacteria. Ambient air contains multiple agents, and exclusive exposure to endotoxin is only found in experimental research. Although all the included studies considered endotoxin exposure as a potential cause of the respiratory outcomes, other possible causative agents were often considered as well and we came across interesting findings regarding other bioaerosol exposures, as shown in Additional file 1: Table S2.

### (Dis)agreement with current scientific literature

The search for a relationship between organic dusts and disease is an ongoing challenge given the inherent aspect of exposure to multiple agents and the difficulty to prove causal relationships in observational epidemiological studies. However, the findings of this review are in line with previous research findings in higher exposed populations. Several occupational studies among farmers have shown increased prevalence of respiratory diseases related to exposure to endotoxin [3–6] and studies experimenting with direct inhalation of endotoxin have shown an inflammatory response in the airways [12]. In addition, Radon et al. investigated the prevalence of respiratory symptoms among inhabitants of rural areas. They found that the number of animal houses in the neighbourhood was a predictor of self-reported wheeze and decreased FEV_1_ [18]. More recently, a Dutch study revealed a relationship between living in the vicinity of a large number of neighbouring farms and lower MMEF values and also between ammonia and particulate air pollution and lower FEV_1_ values, potentially related to endotoxin exposure [2].

From our results it seems that individuals with atopy or a chronic lung disease might be more susceptible to effects of exposure to endotoxin. This is in line with the findings of a study among COPD patients presented by Borlée et al. in 2015 [1]. Here, COPD patients living in the vicinity of livestock farms were found to have more exacerbations and use more medication. More evidence should be sought to confirm that patients with asthma or COPD and atopics form a vulnerable subgroup for the effects of exposure to airborne endotoxin.

Living near a farm was also associated with a *lower* prevalence of allergic rhinitis [1, 63]. Several studies in occupationally exposed farming populations have shown a dual effect of endotoxin with both negative and protective effects, but these populations were exposed to average endotoxin levels above 100 EU/m^3^ [16, 17]. Our focus on lung function and symptoms led to inclusion of studies showing adverse effects of endotoxin exposure. Furthermore, most studies that showed protective effects of endotoxin in homes analyzed endotoxin concentrations in house dust samples, whereas our systematic review only includes airborne endotoxin levels [64, 65].

### Future perspectives

This study adds to the knowledge in the field by summarizing all the evidence available on respiratory effects of exposure to low levels of endotoxin, but future research is needed to strengthen the evidence. Endotoxin is known to originate from rural activities such as farming and composting and adds to air pollution in areas with a high density of these sources. Since possible effects are suggested by this review and other studies, endotoxin in ambient air should be seriously considered and investigated in larger populations. Large studies focussing on long-term exposed individuals are expected to give the best results. Since it is impossible to measure airborne personal exposure for a large group of individuals, modelling of personal exposure seems to be a good way to predict average long-term exposure levels. If a relationship between endotoxin and respiratory complaints becomes more evident, safety measures should be considered in order to protect inhabitants of areas with increased levels of these air pollutants. Another interesting topic for future research would be the effect of exposure to low levels of endotoxin on specific vulnerable subgroups, such as broncho-obstructive patients or atopics, since the respiratory effects seem different in these groups than in the general population.

## Conclusion

Respiratory health effects of exposure to low levels of airborne endotoxin are found in multiple studies. More research regarding this relationship is needed in order to be able to inform/advise neighbouring residents of livestock farms and form guidelines and policies on ambient exposure to endotoxin. Special attention should be given to respiratory effects of endotoxin exposure in vulnerable subgroups, such as patients with broncho-obstructive disease.

## Additional file

Additional file 1: Supplement 1. Pubmed search strategy. Supplement 2. Study incentives and other airborne exposures found to be associated with the outcome variables. **Table S2.** Study incentives and other airborne exposures found to be associated with the outcome variables. Supplement 3: Quality Assessment of included studies. **Table S3.** Quality Assessment of included studies based on the NIH Quality Assessment Tool for Observational Cohort and Cross-Sectional Studies. Supplement 4. Results tables **Table S4.1.** Overview of results – questionnaire outcomes among subjects exposed to bioaerosols. **Table S4.2.** Overview of results – spirometry outcomes among subjects exposed to bioaerosols. **Table S4.3.** Overview of results – exposure-response relationships between endotoxin exposure and respiratory outcomes. **Table S4.4** Analysis of effects of endotoxin exposure on respiratory health in subgroups. Supplement 5: Best evidence synthesis. (DOCX 56 kb)

## Abbreviations

ATS-DLD: American Thoracic Society Division of Lung Disease
COPD: Chronic Obstructive Pulmonary Disease
ECRHS: European Community Respiratory Health Survey
EU: Endotoxin Units
FeNO: Fractional exhaled nitric oxide
FEV_1_: Forced Expiratory Volume, in first second
FVC: Forced Vital Capacity
ISAAC: International Study of Asthma and Allergies in Childhood
LAL: Limulus Amebocyte Lysate
LRT: Lower respiratory tract
MEF: Midexpiratory flow
MMEF: Maximum midexpiratory flow (=FEF_25–75_)
MRC: Medical Research Council
MWF: Metal working fluids
NIH: National Institutes of Health
PEF: Peak expiratory flow
PM: Particulate matter
PRISMA: Preferred Reporting Items for Systematic Reviews and Meta-Analyses
VC: Vital capacity
