# Supplementary material for: Respiratory health effects of exposure to low levels of airborne endotoxin – a systematic review
Source: Environ Health. 2018 Feb 8;17:14. doi: 10.1186/s12940-018-0360-7 (PMC5806377; doi:10.1186/s12940-018-0360-7)
Supplement: Supplementary file 1 — Supplement 1. Pubmed search strategy. Supplement 2. Study incentives and other airborne exposures found to be associated with the outcome variables. Table S2. Study incentives and other airborne exposures found to be associated with the outcome variables. Supplement 3: Quality Assessment of included studies. Table S3. Quality Assessment of included studies based on the NIH Quality Assessment Tool for Observational Cohort and Cross-Sectional Studies. Supplement 4. Results tables Table S4.1. Overview of results – questionnaire outcomes among subjects exposed to bioaerosols. Table S4.2. Overview of results – spirometry outcomes among subjects exposed to bioaerosols. Table S4.3. Overview of results – exposure-response relationships between endotoxin exposure and respiratory outcomes. Table S4.4 Analysis of effects of endotoxin exposure on respiratory health in subgroups. Supplement 5: Best evidence synthesis. (DOCX 56 kb) [file 12940_2018_360_MOESM1_ESM.docx]

Supplementary text

**Supplement 1: Pubmed search strategy**

| **Endotoxin** | Endotoxin  Endotoxins |
| --- | --- |
| **Respiratory symptoms and lung function** | Respiratory symptoms  Asthma  Wheeze  Cough  Dyspnoea  Chest tightness  Lung function  Spirometry  Pulmonary function  FEV1  PEF  FVC  Forced expiratory volume  Forced vital capacity  Spirometer |
| **Exposure** | Exposure*  Exposed  Level*  Concentration* |

**Search query:**

(((endotoxin OR endotoxins) AND ("respiratory symptoms" OR asthma OR wheeze OR cough OR dyspnoea OR "chest tightness" OR "lung function" OR spirometry OR "pulmonary function" OR FEV1 OR PEF OR FVC OR “forced expiratory volume” OR “forced vital capacity” OR spirometer) AND (exposure* OR exposed OR level* OR concentration*)))

Result: 1362 articles

**Search query (including language selection):**

(((endotoxin OR endotoxins) AND ("respiratory symptoms" OR asthma OR wheeze OR cough OR dyspnoea OR "chest tightness" OR "lung function" OR spirometry OR "pulmonary function" OR FEV1 OR PEF OR FVC OR “forced expiratory volume” OR “forced vital capacity” OR spirometer) AND (exposure* OR exposed OR level* OR concentration*))) AND (English[Language] OR Dutch[Language] OR German[Language] OR French[Language])

Result: 1322 articles

**Supplement 2: Study incentives and other airborne exposures found to be associated with the outcome variables.**

Table S2. Study incentives and other airborne exposures found to be associated with the outcome variables.

| **Study** | **Study incentives and other exposures associated with outcome.** |
| --- | --- |
| **Kateman et al. (1990)** | This study was initiated after several cases of an influenza-like illness occurred among workers after working in an area with a specific cold-water spray humidifier. |
| **Dahlqvist et al. (1992)** | This study focussed on effects of exposure to moulds and significant findings were found among the subjects proven to be sensitive to moulds (precipitating antibodies positive). |
| **Sprince et al. (1997)** | Significant exposure-response relationships were found between respiratory symptoms and total aerosol, culturable fungi and bacteria. |
| **Mahar et al. (2002)** | Concerns about the health impact of processing solid waste into refuse-derived fuel led to a health records study in 1995. This study represents continuation of the earlier study in 1995. |
| **Heldal et al. (2004)** | A significant association between exposure to fungal spores and cough was found. |
| **Sigsgaard et al. (2004)** | Workers in this plant suffered from a range of respiratory symptoms after changing to recycled paper. In 1994, evidence was found that symptoms were related to endotoxin exposure in a dose-dependent manner. |
| **Rabinovitch et al. (2005)** | Measurements were performed at a school for children with significant asthma that interferes with regular school attendance and progress. |
| **Rusca et al. (2008)** | The analysis of the association between prevalence of self-reported bronchial syndrome and airborne fungi concentration revealed a strong positive dose-response relationship. |
| **Dang et al. (2010)** | After opening of this waterpark, eye and respiratory irritation symptoms were increasingly reported. Suspicion of trichloramine and endotoxin as a cause. |
| **Heldal et al. (2015)** | Significant associations between exposure to actinomycetes and work-related nasal and cough symptoms and lung function decline was found. |

# **Supplement 3: Quality Assessment of included studies**

Table S3. Quality Assessment of included studies based on the NIH Quality Assessment Tool for Observational Cohort and Cross-Sectional Studies.(20)

| **Author (year)** | **Research question** | **Study**  **population** | **Participation rate >50%** | **Recruitment of subjects** | **Effect size** | **Exposure prior to outcomes** | **Time frame** | **Different levels of exposure** | **Exposure definition** | **Exposure assessment over time** | **Outcome definition** | **Blinding** | **Loss to follow up ≤20%** | **Confounding** | **Quality rating** | **Quality rating percentage** | **Quality rating score** |
| --- | --- | --- | --- | --- | --- | --- | --- | --- | --- | --- | --- | --- | --- | --- | --- | --- | --- |
| **Kawamoto et al. (1987)** | + | + | + | + | + | NA | + | + | + | - | + | - | NA | + | 10/12 | 83% | M |
| **Kateman et al. (1990)** | + | - | NR | + | + | NA | + | - | + | + | + | - | NA | + | 8/12 | 67% | W |
| **Dahlqvist et al. (1992)** | + | - | NR | + | + | NA | + | - | + | + | + | - | NA | + | 8/12 | 67% | W |
| **Sprince et al. (1997)** | + | + | + | + | + | NA | + | + | + | - | + | - | NA | + | 10/12 | 83% | M |
| **Zock et al. (1998)** | + | + | + | + | + | NA | + | + | + | + | + | - | NA | + | 11/12 | 92% | S |
| **Mandryk et al. (1999)** | + | + | + | + | + | NA | + | + | + | + | + | - | NA | + | 11/12 | 92% | S |
| **Mahar et al. (2002)** | + | + | NR | + | + | + | + | - | + | + | + | - | NR | - | 9/14 | 64% | W |
| **Wouters et al. (2002)** | + | + | NR | + | + | NA | NA | - | + | + | + | - | NA | NR | 7/11 | 64% | W |
| **Fransman et al. (2003)** | + | + | + | + | + | NA | NA | + | + | + | + | - | NA | + | 10/11 | 91% | S |
| **Heldal et al. (2004)** | + | - | NR | + | - | NA | NA | - | + | + | + | - | NA | + | 6/11 | 55% | W |
| **Kennedy et al. (2004)** | + | + | + | + | + | NA | + | + | + | + | + | - | + | + | 12/13 | 92% | S |
| **Sigsgaard et al. (2004)** | + | + | + | + | + | + | + | + | + | + | + | - | - | + | 12/14 | 86% | M |
| **Rabinovitch et al. (2005)** | + | + | NR | + | + | NA | NA | + | + | + | + | + | NA | - | 9/11 | 82% | M |
| **Schiffman et al. (2005)** | + | + | + | + | + | NA | + | - | + | - | + | - | NA | NR | 8/12 | 67% | W |
| **Smit et al. (2005)** | + | + | + | + | + | NA | NA | + | + | + | + | - | NA | + | 10/11 | 91% | S |
| **Hoopmann et al. (2006)** | + | + | + | + | + | NA | NA | + | + | + | + | + | NA | + | 11/11 | 100% | S |
| **Horick et al. (2006)** | + | + | + | + | + | + | + | + | + | + | + | + | + | + | 14/14 | 100% | S |
| **Widmeier et al. (2007)** | + | + | + | + | + | NA | NA | - | + | + | + | - | NA | + | 9/11 | 82% | M |
| **Rusca et al. (2008)** | + | + | + | + | + | NA | NA | + | + | - | + | - | NA | + | 9/11 | 82% | M |
| **Dang et al. (2010)** | + | + | NR | + | + | NA | NA | - | + | - | - | - | NA | + | 6/11 | 55% | W |
| **Renström et al. (2011)** | + | - | + | + | + | NA | NA | - | + | + | + | - | NA | + | 8/11 | 72% | M |
| **Schlunssen et al. (2011)** | + | + | + | + | + | NA | NA | + | + | - | + | - | NA | + | 9/11 | 82% | M |
| **Meza et al. (2013)** | + | - | + | + | + | NA | NA | - | + | - | + | - | NA | + | 7/11 | 64% | W |
| **Ramagopal et al. (2014)** | + | + | NR | + | + | NA | NA | + | + | - | + | + | NA | + | 9/11 | 82% | M |
| **Shiryaeva et al. (2014)** | + | + | + | + | + | NA | + | + | + | + | + | - | NA | + | 11/12 | 92% | S |
| **Cyprowski et al. (2015)** | + | - | NR | + | + | NA | + | + | + | + | + | - | NA | + | 9/12 | 75% | M |
| **Delfino et al. (2015)** | + | + | NR | + | + | NA | NA | + | + | + | + | + | + | + | 11/12 | 92% | S |
| **Heldal et al. (2015)** | + | - | + | + | + | NA | + | + | + | + | + | - | NA | + | 10/12 | 83% | M |
| **Lai et al. (2015)** | + | + | + | + | + | NA | + | + | + | - | + | + | - | + | 11/13 | 85% | M |
| **Bose et al. (2016)** | + | + | + | + | + | + | + | + | + | - | + | + | NR | + | 12/14 | 86% | M |
| **Ghani et al. (2016)** | + | + | NR | + | + | NA | NA | - | + | - | + | - | NA | + | 7/11 | 64% | W |

Quality rating score: ≥90% = strong (S), 70-90% = moderate (M), <70% = weak (W)

# **Supplement 4. Results tables**

Table S4.1. Overview of results – questionnaire outcomes among subjects exposed to bioaerosols.

| **Results Questionnaire** | | | |
| --- | --- | --- | --- |
| **Author (year)** | **Population** | **Levels of airborne endotoxin exposure (EU/m³)** | **Conclusion** |
| ***Non-occupational populations*** | | | |
| **Schiffman et al. (2005)** | 48 healthy subjects. | 7.40 EU/m³ | No significant difference in the prevalence of cough between experimental (exposed) and control (unexposed) measurements. |
| **Hoopmann et al. (2006)** | 3867 children. | Median 0.064 EU/m³, IQR 0.025-0.141* | 15.9% of the boys and 12.9% of the girls reported wheezing in the last 12 months. Wheezing, nocturnal coughing and asthmatic symptoms were more prevalent in children of atopic parents (all p<0.001). |
| ***Occupational populations*** | | | |
| **Kateman et al. (1990)** | 40 textile yarn workers exposed to spray-humidifier, 42 controls exposed to other or no humidifier. | GM 0.64 EU/m³ (GSD 0.016) for cold-water humidification area, 0.18-0.19 EU/m³ for other areas. | 25% of workers complained of wheezing, no differences between the subpopulations. |
| **Dahlqvist et al. (1992)** | 28 wood trimmers, 19 controls (office workers) | 15-25 EU/m³.* | Compared to the controls, wood trimmers had a **significantly** higher prevalence of dry cough (p=0.002), cough with phlegm (p=0.038) and breathlessness (p=0.020). |
| **Sprince et al. (1997)** | 183 machine workers in automobile industry.  66 assemblers(controls). | GM 31 EU/m³, range 2.7-984 | Symptoms reported **significantly** more often by subjects than by controls were: cough (OR 3.1; 95% CI 1.4-6.9); phlegm (OR 3.1; 95% CI 1.6-6.1); work-related chest-tightness (OR 5.9; 95% CI 1.4-25.7); post-shift chest tightness (OR 4.5; 95% CI 1.3-15.2) and post-shift cough (OR 4.0; 95% CI 1.2-14.1). |
| **Zock et al. (1998)** | 57 potato processing workers. | AM 32.9 EU/m³.  Low exposed group: AM 21 EU/m³, high exposed group: 56 EU/m³. | 25% of the exposed workers reported at least one of the work-related respiratory symptoms (cough, phlegm, shortness of breath, chest tightness). These seemed to be more prevalent among the low exposed group. |
| **Mandryk et al. (1999)** | 168 wood workers. 30 maintenance workers (controls). | Inhalable endotoxin: GM 24.1-43.0 EU/m³(GSD 15.5-47.7). Respirable endotoxin: GM 3.3-6.5 EU/m³(GSD 13.5-41.0).* | Regular cough and phlegm were **significantly** more prevalent in the woodworkers when compared to controls, 61% vs 23.5% and 61.5% vs 23.5%(p<0.001). Chronic bronchitis was also more prevalent in woodworkers (30.3%) compared to controls (11.8%) (p<0.05). |
| **Wouters et al. (2002)** | 47 waste collecting workers. 15 controls. | GM 39.4 EU/m³,  range 4-7182 | No significant difference in prevalence of respiratory symptoms among exposed workers when compared to controls. |
| **Fransman et al. (2003)** | 112 plywood mill workers. 415 controls of the general population. | GM 23.0 EU/m³ (GSD 2.8) | **Significantly** more attacks of shortness of breath with wheezing were found in subjects, OR 1.8 (95%CI 1.0-3.1, p<0.05). Almost all other asthma symptoms were increased in the exposed group, but did not reach significance. Shortness of breath with wheezing, waking by shortness of breath and asthma were **significantly** more present in workers employed >6.5 years (all p<0.05). 53-83% of respiratory symptoms lessened during holidays. |
| **Heldal et al. (2004)** | 22 waste collection workers. | AM 2.5 EU/m³,  range 0 -7.8 | 58% of the workers reported any work-related symptom, 41% reported any respiratory symptom, 15% reported cough. No statistically significant difference in exposure level to endotoxin between subjects with and without symptoms. |
| **Kennedy et al. (2004)** | 226 glass bottle recycling workers. 212 ferry workers (controls). | GM 3.6-4.3 EU/m³,  range <0.14-179 | Attacks of chest tightness were **significantly** more prevalent in the exposed groups than in the control group: 22-41% in subjects vs 17% in controls (OR 1.8, 95%CI 1.1-3.3). Chest tightness at work was also more prevalent in the exposed groups than in the control group: 5-15% in subjects vs 2% in controls (OR 4.8, 95%CI 1.1-21.5). |
| **Smit et al. (2005)** | 371 waste water workers. 97 office staff, 2698 general population members (controls). | GM 27 EU/m³ (GSD 3.7) | Prevalence of daily cough (PR 1.48; 95%CI 1.20-1.82), shortness of breath (PR 1.48; 95%CI 1.04-2.09) and asthma attacks (PR 1.79; 95%CI 1.24-2.59) were **significantly** higher among exposed subjects than in the general population. Length of employment >20 years was **significantly** associated with lower respiratory and skin symptoms (PR 6.58; 95% CI 2.49-17.35; p<0.001). |
| **Widmeier et al. (2007)** | 409 wastewater-and garbage workers. 369 public transport and forestry workers (controls). | Waste water workers: winter 8.8-29.7 EU/m³, summer 29.8-52.6 EU/m³; garbage collectors winter 3.43-8.14 EU/m³, summer 3.63-11.03 EU/m³. | Respiratory symptoms were not more prevalent in exposed subjects when compared to controls. |
| **Rusca et al. (2008)** | 111 sawmill workers | Range 1-24 EU/m³ | No significant relationship between the medical complaints and the concentrations of endotoxin or dust. |
| **Dang et al. (2010)** | 69 water resort workers, 74 office workers(controls). | Mean 45 EU/m³,  range 18-84 | Exposed subjects had **significantly** more work-related symptoms than unexposed: cough PR 10.24 (95% CI 4.33-24.23), wheezing PR 9.74 (95% CI 2.36-40.19), shortness of breath PR 6.70 (95% CI 2.47-18.20), chest tightness PR 6.67 (95% CI 2.08-21.35). Symptoms of cough were **significantly** increased on high occupancy days (>1000 patrons) compared with low occupancy days (<100 patrons), PR 2.23 (95%CI 1.10-4.52). |
| **Renström et al. (2011)** | 59 pet shop workers. | Range 1-100 EU/m³ | 22% reported wheezing or chest tightness. No significant difference in exposure levels of endotoxin between subjects with work symptoms compared to subjects without symptoms. |
| **Schlunssen et al. (2011)** | 232 woodchip and straw workers. 107 oil/gas workers (controls). | Woodchip plants: median 1.7 EU/m³, range 0.01-6.5.  Straw plants: median 74 EU/m³, range 1.5-294.  Control: median 0.9 EU/m³ | Asthma symptoms were significantly more prevalent in straw workers when compared to controls (OR 7.6; 95%CI 1.4-40.4). |
| **Meza et al. (2013)** | 183 aircraft workers exposed to MWF. 224 office workers (controls). | Mean 1.2 EU/m³,  range 0.42-2.7 | Among the exposed workers, wheezing was **significantly** more present when compared to the controls, PR 1.54 (95%CI 1.03-2.29). Also, decline in wheezing symptoms on vacation/days off was **significantly** greater in the exposed group, PR 2.84 (95%CI 1.56-5.18). Exposed workers woke **significantly** more often with a feeling of chest tightness (PR 2.4; 95%CI 1.30-4.69) and this also improved **significantly** more on vacation days when compared to controls (PR 2.22; 95%CI 1.05-4.72). Work related asthma symptoms were **significantly** more prevalent among the exposed workers, PR 1.92 (95%CI 1.19-3.09). |
| **Shiryaeva et al. (2014)** | 70 salmon processing workers. | Monday-Thursday GM 1.39-1.65 EU/m³, range 0.30-29.0 | The highest frequency of symptoms was seen on Mondays and decreased gradually over the week. Wheeze and chest tightness decreased **significantly** over the workweek (p<0.05). |
| **Heldal et al. (2015)** | 47 compost workers, 37 office controls | AM 4.0-38 EU/m³,  range 0-730 | Cough (OR 4.3, 95%CI 1.0-18.2) and one or more work-related symptoms (OR 4.0, 95%CI 1.1-14.7) were **significantly** more prevalent in the compost workers when compared to controls. |

* original values were presented in article in mg/m³ or ng/m³

Table S4.2. Overview of results – spirometry outcomes among subjects exposed to bioaerosols.

| **Results Spirometry** | | | |
| --- | --- | --- | --- |
| **Author (year)** | **Population** | **Levels of airborne endotoxin exposure (EU/m³)** | **Conclusion** |
| ***Non-occupational populations*** | | | |
| **Schiffman et al. (2005)** | 48 healthy subjects. | 7.40 EU/m³ | No significant difference between change in lung function (FEV_1_, FVC and FEF25-75%) parameters after 1 hour exposure to 7.40 EU/m³ airborne endotoxin when compared to control group (p>0.80). |
| **Delfino et al. (2015)** | 43 asthmatic school-children. | Mean 2.04 EU/m³ (±3.71),  range 0.002-25.3 | Subjects with low FEV_1_ (<80% predicted) had a **nominally significant** association with lag one (p<0.06) and a **significant** association with 2-day average endotoxin exposure (p<0.005). Subjects with low FEV₁ (<80% predicted) experienced a **significant** 7.7% drop in %predicted FEV₁ (95%CI -12.3% to  -3.3%), while those with higher FEV₁ values showed a non-significant 0.5% drop (95%CI -2.2% to 1.2%). |
| ***Occupational populations*** | | | |
| **Kawamoto et al. (1987)**  **US** | 128 cotton workers. | 3 groups: <17 EU/m³, 17-117 EU/m³ and >117 EU/m³* | 13% of subjects had >5% decrements in FEV_1_ over the work shift. No statistically significant differences in baseline lung functions between workers employed for different lengths of time. |
| **Kateman et al. (1990)** | 40 textile yarn workers exposed to spray-humidifier, 42 controls exposed to other or no humidifier. | GM 0.64 EU/m³ (GSD 0.016) for cold-water humidification area, 0.18-0.19 EU/m³ for other areas. | In the workers of the cold-water department the mean change in FEV_1_ was -0.096 L (SD 0.159) on Day 1, -0.065 L (SD 0.134) on Day 2, -0.067 L (SD 0.130) on Day 3 **(all p<0.01)**. FVC, MMEF, MEF25, MEF50 and MEF75 also showed significant decline across the work shift and over the week in this group. The lung function level on Friday was reduced when compared to Monday. The other workers did not show a significant decline in lung function. |
| **Dahlqvist et al. (1992)** | 28 wood trimmers, 19 controls (office workers | 15-25 EU/m³.* | There were no significant differences between the lung function values in wood trimmers and controls. Wood trimmers sero–positive for precipitating antibodies to moulds showed a **significant** decrease in FEV_1_ over a workweek. Subjects with a period of employment >18 years had a significantly larger change in MEF over the workweek than subjects employed <6 years. |
| **Sprince et al. (1997)** | 183 machine workers in automobile industry. 66 assemblers (controls). | GM 31 EU/m³,  range 2.7-984 | Pre-shift %predicted FEV₁ and FVC did not differ significantly. Machine operators did not significantly differ from controls in proportion who decreased >5% for FEV_1_ (16% vs 12%, p=0.42) or FVC (14% vs 9%, p=0.33). |
| **Zock et al. (1998)** | 57 potato processing workers. | AM 32.9 EU/m³.  Low exposed group: AM 21 EU/m³, high exposed group: 56 EU/m³. | Baseline lung function seemed to be lower in the high exposed group, although this did not reach statistical significance. Mean FEV_1_ and MMEF showed a **significant** decrease across each shift. Mean absolute decrease in FEV₁ was 0.06-0.12 L, mean absolute decrease in FVC was 0.16-0.28 L/sec (both p<0.01). |
| **Mandryk et al. (1999)** | 168 wood workers. 30 maintenance workers (controls). | Inhalable endotoxin: GM 24.1-43.0 EU/m³(GSD 15.5-47.7).  Respirable endotoxin: GM 3.3-6.5 EU/m³(GSD 13.5-41.0).* | Woodworkers had **significantly** lower predicted lung functions than controls. VC 83.45 ± 3.12 vs 95.35 ± 7.93 (p=0.0001), FVC 84.66 ± 0.72 vs 94.90 ± 3.85 (p=0.0001), FEV_1_ 84.67 ±1.20 vs 93.11 ± 2.81 (p=0.0001). The cross-shift decrease in lung function was **significant** for all lung function parameters (p<0.001) in woodworkers compared with controls (VC 6.32% vs 2.37% decrease; FVC 4.40% vs 2.12% decrease; FEV₁ 6.34% vs 1.78% decrease; FEV₁/FVC 2.86% vs 0.06% decrease; FEF25-75% 7.74% vs 2.22% decrease; PEF 6.62% vs 5.20% decrease). Percentage predicted lung function was positively correlated with the number of years exposed to wood dust. |
| **Mahar et al. (2002)** | 87 refuse derived fuel workers. | 1995: GM 28.5 EU/m³ (GSD 2.77). 2000 GM 28.1 EU/m³ (GSD 6.65). Total GM 28.4 EU/m³ (GSD 3.75). | No significant changes in lung function over the period of 1995-2000 in exposed workers. ∆FVC 1995-2000: mean -0.76 (SD 11.9); p=0.33. ∆FEV_1_ mean -1.31(SD 12.5); p=0.76. No significant differences between workers employed less than 10 years and workers employed more than 10 years in decrease in lung function. |
| **Sigsgaard et al. (2004)** | 97 paper mill workers. 55 water-supply workers (controls). | 6-69 EU/m³,  range 6-370. 3 groups: low, medium and high exposed. | Loss of lung function in ml/year did not differ significantly among the groups, ranging from -36.62 ml/year to -54.09 ml/year for FEV_1_. |
| **Widmeier et al. (2007)** | 409 wastewater-and garbage workers. 369 public transport and forestry workers (controls). | Waste water workers: winter 8.8-29.7 EU/m³, summer 29.8-52.6 EU/m³; garbage collectors winter 3.43-8.14 EU/m³, summer 3.63-11.03 EU/m³. | A statistical significant difference in FEV_1_ and FVC among garbage collectors when compared to the other groups was seen only in current smokers and disappeared after considering smoking in the model. |
| **Rusca et al. (2008)** | 111 sawmill workers. | Range 1-24 EU/m³ | Lung function tests were not significantly associated with exposure to bioaerosols. |
| **Renström et al. (2011)** | 59 pet shop workers. | Range 1-100 EU/m³ | Lung function values were on average slightly lower than expected among the exposed: 91.4% (SD 10.9) for VC, 93.3% (SD 11.1) FVC, 94.8% (SD 13.3) for FEV_1_. |
| **Shiryaeva et al. (2014)** | 70 salmon processing workers. | Monday-Thursday GM 1.39-1.65 EU/m³, range 0.30-29.0 | **Significant** decline in cross-shift %FEV_1_ was seen on Monday with a mean decrease of 0.41-0.93 % FEV₁ (SD 5.24-7.52) (p<0.05). Analysis of cross-week change in FEV₁ did not show significant change. |
| **Cyprowski et al. (2015)** | 78 sewage workers. | AM 38.8 EU/m³,  range 0.63-214 | Mean values of FEV_1_ and FVC declined during work shift; FVC 4.64 vs 4.60, FEV₁ 4.00 vs 3.98 (l). |
| **Heldal et al. (2015)** | 47 compost workers, 37 office controls | AM 4.0-38 EU/m³,  range 0-730 | Predicted FVC% measured before work was **significantly** lower in the compost workers as compared to controls (p<0.05). Cross-shift changes in lung function parameters were not significantly different between subjects and controls. |
| **Ghani et al. (2016)** | 100 textile mill workers, 100 controls. | Range 40-300EU/m³ | The mean values of FVC, FEV_1_, FEV₁/FVC and PEF were **significantly** reduced in the exposed subjects of all age groups compared to a control group (p<0.05). This effect is highly significant in the age group 30-40 years (p<0.001) |

* original values were presented in article in mg/m³ or ng/m³

Table S4.3. Overview of results – exposure-response relationships between endotoxin exposure and respiratory outcomes.

| **Results Exposure-response analysis** | | | | |
| --- | --- | --- | --- | --- |
| **Author (year)** | **Population** | | **Levels of airborne endotoxin exposure (EU/m³)** | **Dose-dependent effects** |
| ***Non-occupational populations*** | | | | |
| **Rabinovitch et al. (2005)** | | 24 asthmatic school-children | Interval 1: median 0.08 EU/m³, IQR 0.09.  Interval 2: median 0.37 EU/m³, IQR 0.16. | Evening FEV_1_ levels were **significantly** lower when children were exposed to higher personal levels of endotoxin, -316ml (95%CI -597 to -36; p=0.036) per 1 EU/m³ increase in personal endotoxin exposure.  For every increase of 1 EU/m³ endotoxin exposure, the OR for sleep-related asthma symptoms was **2**.**042 (95%CI 1.029-4.042; p=0.041).** |
| **Hoopmann et al. (2006)** | | 3867 children | Median 0.064 EU/m³, IQR 0.025-0.141* | Per one unit log increase in endotoxin exposure the OR for asthmatic symptoms among children with atopic parents was **1.15 (95%CI 1.03-1.29, p=0.0156)**. For children of non-atopic parents, the OR was 0.95 (95%CI 0.88-1.05). For children with atopic parents, wheezing had an OR of **1.15 (95%CI 1.04-1.27)** per one unit increase of log endotoxin. |
| **Horick et al. (2006)** | | 360 children | Mean 0.81 EU/m³,  range 0.23-5.87 | An increase of 0.39 log_10_(EU/m³) in airborne endotoxin exposure resulted in a **significant** increase in prevalence of wheeze, with a relative risk of **5.56 (95%CI 1.19-26.03).** |
| **Ramagopal et al. (2014)** | | 75 children | SIM: median 0.6 EU/m³, range 0.03 - 8.6.  PIPER: median 1.0 EU/m³, range 0.09-16. | The OR for wheeze for children exposed to 0.57-1.03 EU/m³ was 3.0 (95%CI 0.6-14.8) when compared to children exposed to lower levels of endotoxin when measured with PIPER; for SIM, this OR was 1.3 (0.3-6.1). For asthma, the OR children exposed to 0.57-1.03 EU/m³ was 2.6 (95%CI 0.6-11.3) when measured with PIPER and 1.5 (95%CI 0.4-6.0) when measured with SIM. |
| **Delfino et al. (2015)** | | 43 asthmatic school-children | Mean 2.04 EU/m³ (±3.71),  range 0.002-25.3 | An increase of 2.19 EU/m³ resulted in a **significant** 7.7% (95%CI -12.3 to -3) drop in %predicted FEV₁ in patients with a baseline %predicted FEV_1_ <80%. |
| **Lai et al. (2015)** | | 248 asthmatic school-children | GM 24.7 EU/m³,  range 0.2-780.0  (of which 78% <90 EU/m³) | An increase in airborne endotoxin from 0.2 EU/m³ to 24.7 EU/m³ was associated with a dose-dependent increase in maximum asthma symptoms days for children with non-atopic asthma, adjusted IRR 1.16 (95%CI 1.03-1.31); atopic asthma 1.00 (0.93-1.07). Maximum asthma symptoms days increased with 1.3 days for each 14-day period. Daytime wheeze and exercise-related symptoms **significantly** increased when exposure levels increased from 0.2 EU/m³ to 24.7 EU/m³; IRR 1.21 (95%CI 1.06-1.38) and IRR 1.45 (1.19-1.77) respectively. |
| **Bose et al. (2016)** | | 84 COPD patients | Mean 0.55 EU/m³ (±1.3) | Per 1 unit increase in log_10_ airborne endotoxin, the OR for nocturnal symptoms was 1.06 (95%CI 0.74-1.52; p=0.74) and the OR for severe exacerbations was 0.66 (95%CI 0.35-1.25; p=0.20). |
| ***Occupational populations*** | | | | |
| **Kawamoto et al. (1987)** | 128 cotton workers. | | 3 groups: <17 EU/m³, 17-117 EU/m³ and >117 EU/m³* | The mean percentage change in FEV_1_ over the work shift was -1.09 for the group exposed to 17-117 EU/m³ and -1.27 for the group exposed to more than 117 EU/m³, these decreases did not reach statistical significance. |
| **Sprince et al. (1997)** | 183 machine workers. 66 controls. | | GM 31 EU/m³, range 2.7-984 | Usual phlegm showed a **significant** association with a one log increase in endotoxin exposure, OR 1.24 (95% CI 1.04-1.47). |
| **Zock et al. (1998)** | 57 potato processing workers. | | AM 32.9 EU/m³.  Low exposed group: AM 21 EU/m³, high exposed group: 56 EU/m³. | Across-shift decrease in FEV_1_, FVC and MMEF was **significantly larger (p<0.01)** in the high exposed group than in the low exposed group. |
| **Mandryk et al. (1999)** | 168 wood workers. 30 maintenance workers (controls). | | Inhalable endotoxin: GM 24.1-43.0 EU/m³(GSD 15.5-47.7).* | Per increase in one log unit of endotoxin exposure, FEV_1_ decreased with **0.33 (p<0.001), FVC with 0.30 (p<0.001)**. Per increase in one unit of endotoxin exposure, the cross-shift change in **VC was -0.32 (p<0.001).** |
| **Kennedy et al. (2004)** | 226 glass bottle recycling workers. 212 ferry workers (controls). | | GM 3.6-4.3 EU/m³,  range <0.14-179 | There were no significant increases found in upper airway symptoms (OR 1.6, 95%CI 0.7-3.2), chest symptoms (OR 1.5, 95%CI 0.6-4.7) or somatic symptoms (OR 1.6, 95%CI 0.6-4.4) when related to personal endotoxin exposure > 4 EU/m³ vs <4 EU/m³. |
| **Sigsgaard et al. (2004)** | 97 paper mill workers. 55 water-supply workers (controls). | | 6-69 EU/m³,  range 6-370. 3 groups: low, medium and high exposed. | Exposure to endotoxin was negatively associated with FVC decline, **significantly** so for the highest exposed group of paper workers with B 23.4 (95%CI 5.9-41.0, p=0.009). |
| **Smit et al. (2005)** | 371 waste water workers. 97 office staff, 2698 general population (controls). | | GM 27 EU/m³ (GSD 3.7).  3 groups: <50, >50-200, >200 EU/m³ | No statistically significant increase in respiratory symptoms with increasing levels of exposure. |
| **Schlunssen et al. (2011)** | 232 woodchip and straw workers. 107 workers in oil/gas power plants (controls). | | Woodchip plants: median 1.7 EU/m³, range 0.01-6.5.  Straw plants: median 74 EU/m³, range 1.5-294.  Control: median 0.9 EU/m³ | Increased asthma symptoms were **significantly** associated with exposure to endotoxin in the most exposed group (12.6-294 EU/m³); adjusted OR 8.1 (1.5-44.4). In the least and moderate exposed group, this association was not significant; adjusted OR 2.6 (0.3-20.0) for least exposed and 2.9 (0.5-18.7) for the moderate exposed group. No significant relationship was found between level of exposure to endotoxin and lung function parameters. |
| **Shiryaeva et al. (2014)** | 70 salmon processing workers | | Monday-Thursday GM 1.39-1.65 EU/m³, range 0.30-29.0 | Multiple logistic regression showed non-significant increases in prevalence of cough, OR 1.07 (95%CI 0.7-2.04); wheezing OR 1.06 (95%CI 0.02-7.63) and chest tightness, OR 1.03 (95%CI 0.20-4.36) when exposure of endotoxin increased. Change in FEV_1_ in relation to increase in endotoxin exposure was also not statistically significant. |
| **Cyprowski et al.(2015)** | 78 sewage workers. | | AM 38.8 EU/m³,  range 0.63-214 | A **significant** impact on the across shift decline in FEV_1_ was found in the multifactor model; linear regression showed a decrease of 0.042 L in FEV_1_ (p=0.044). |
| **Heldal et al. (2015)** | 47 compost workers, 37 office controls | | AM 4.0-38 EU/m³, range 0-730 | Subjects exposed to 0.7-2.7 EU/m³ endotoxin had a **significant** OR of 4.7 (95%CI 1.2-19) for cough symptoms, as compared to non-exposed controls. For subjects exposed to 2.9-310 EU/m³, the OR for cough was 3.4 (95%CI 0.9-13). |

* original values were presented in article in mg/m³ or ng/m³

Table S4.4 Analysis of effects of endotoxin exposure on respiratory health in subgroups.

| **Subgroup analyses** | | | | | | |
| --- | --- | --- | --- | --- | --- | --- |
| ***Smoking*** | | | | | | |
| **Author (year)** | **Distribution of smokers*** | | | |  | **Effect of smoking** |
| **Dahlqvist et al. (1992)** | 18% ex-smokers, 75% non-smokers, 7% smokers | | | |  | No differences in the distribution of symptoms between smokers and non-smokers. |
| **Zock et al. (1998)** | smokers 55-57%, ex-smokers 22-25%. | | | |  | Non-smokers showed larger across shift declines than smokers, for FEV_1_ the across shift difference was -0.1% (95%CI -3.6;3.5) for smokers and -1.8% (95%CI -4.5;1.0) for non-smokers. |
| **Mahar et al. (2002)** | 34-43% smokers, 57-66% ex- and non-smokers | | | |  | Smokers showed an across shift decline of 1.12% (SD 9.5) for FVC and 2.26% (SD 12.1) for FEV_1_. Non-smokers showed an across shift decline of 0.53% (SD 11.9) for FVC and 0.73% (SD 12.5) for FEV₁. |
| **Sigsgaard et al. (2004)** | S: 26%, C: 58% | | | |  | The lung function decline was comparable among smokers and non-smokers. |
| **Widmeier et al. (2007)** | Waste water: 39% smokers, 29% ex-smokers, 32% never smokers. Garbage workers: 48% smoker, 31% ex-smoker, 21% never smoker.  C: 34% current smokers, 23% ex smokers, 43% never smokers. | | | |  | Exposed smoking garbage workers had significantly lower lung function values than non-exposed smokers. For ex-smokers, no significant difference was found according to exposure. |
| **Schlunssen et al. (2011)** | 28.7-39.9% in subjects, 31.8% in controls. | | | |  | Asthma symptoms were associated with endotoxin in non-smokers (OR10.1;1.7-59.7), whereas this was not found for smokers (OR 0.5; 0.1-2.8) |
| **Shiryaeva et al. (2014)** | 37.1% smokers, 32.9% ex-smokers, 30% never smokers | | | |  | For smokers, mean cross shift decline in FEV_1_ was 0.93% (SD 5.24), this was 0.72% (SD 6.31) for former smokers and 0.41% (SD7.52) for non-smokers. |
| ***Atopics/asthmatics*** | | | | | | |
| **Author (year)** | | **Atopics** | **Asthma/COPD** | **Effect of atopy/asthma** | | |
| **Hoopmann et al. (2006)** | | 19.2% boys, 14.1% girls | 10.9% boys, 14.9% girls | Increase of asthmatic symptoms and wheezing due to exposure to airborne endotoxin was significant for children of atopic parents, but not for children of non-atopic parents. | | |
| **Dang et al. (2010)** | | S: 34%, C: 39% | S:17%, C:12% | 60% of the exposed asthmatic workers reported that their asthma seemed worse at work, while none of the non-exposed asthmatic subjects reported this. | | |
| **Renström et al. (2011)** | | 29% atopic | 7-22% asthmatic | Atopic subjects had a significant higher proportion with symptoms at work (PR 3.2 (95%CI 1.6-6.2), p<0.001) | | |
| **Shiryaeva et al. (2014)** | | 21.4% atopic | 7.1% asthmatic | There were no significant differences in respiratory outcomes related to exposure between atopic and non-atopic subjects. | | |
| **Delfino et al. (2015)** | | - | 100% asthmatic | One study among asthmatic school children found that subjects with baseline FEV_1_ <80% of predicted had significant interactions with endotoxin exposure, predicted FEV₁ values dropped with 7.7% (95%CI -12.3 to -3.3%) for every 2.19 EU/m³ increase in exposure. | | |
| **Lai et al. (2015)** | | 69% atopic | 100% asthmatic | Airborne endotoxin was associated with increased maximum symptom-days only in subjects with non-atopic asthma. For atopics, there was an inverted U-shaped relationship between school air endotoxin and maximum symptom-days (plateau at 230 EU/m³) | | |

* S=subjects, C=controls

**Supplement 5: Best evidence synthesis**Consistent findings = 75% of the findings are significant in one direction

Strong evidence = consistent findings in 2 or more high quality studies
Moderate evidence = consistent findings in 1 high quality study and at least one low quality study; or consistent findings in multiple low quality studies
Weak evidence = no consistent findings, all studies show results in one direction (at least 2 significant) and no conflicting findings exist.
Insufficient evidence = only one study available or inconsistent findings in multiple studies

| **Effect of endotoxin exposure** | **Evidence in included studies** | **Evidence rating** |
| --- | --- | --- |
| **Wheeze** | Worse when exposed (sign):  2 strong (Hoopmann 2006, Horick 2006), 1 moderate (Lai 2015) | Weak evidence |
|  | Worse when exposed (not sign):  1 strong (Shiryaeva 2014), 3 moderate (Ramagopal 2014, Bose 2016, Schlünssen 2011) |  |
|  | Better when exposed (sign): - |  |
|  | Better when exposed (not sign): - |  |
| **Cough** | Worse when exposed (sign): 1 weak (Heldal 2004) | Insufficient evidence |
|  | Worse when exposed (not sign): 1 strong (Shiryaeva 2014) |  |
|  | Better when exposed (sign): - |  |
|  | Better when exposed (not sign): 1 strong (Zock 1998) |  |
| **(nocturnal) Asthma symptoms** | Worse when exposed (sign):  1 strong(Hoopmann 2006), 3 moderate (Rabinovitch 2005, Lai 2015, Schlünssen 2011) | Weak evidence |
|  | Worse when exposed(not sign):  2 moderate (Ramagopal 2014, Bose 2016) |  |
|  | Better when exposed (sign): - |  |
|  | Better when exposed(not sign): - |  |
| **FEV_1_** | Worse when exposed (sign):  4 strong (Delfino 2015, Zock 1998, Mandryk 1999, Cyprowski 2015), 1 moderate (Rabinovitch 2005) | Weak evidence |
|  | Worse when exposed(not sign):  1 strong (Shiryaeva 2014), 1 moderate (Kawamoto 1987) |  |
|  | Better when exposed (sign): - |  |
|  | Better when exposed(not sign): - |  |
